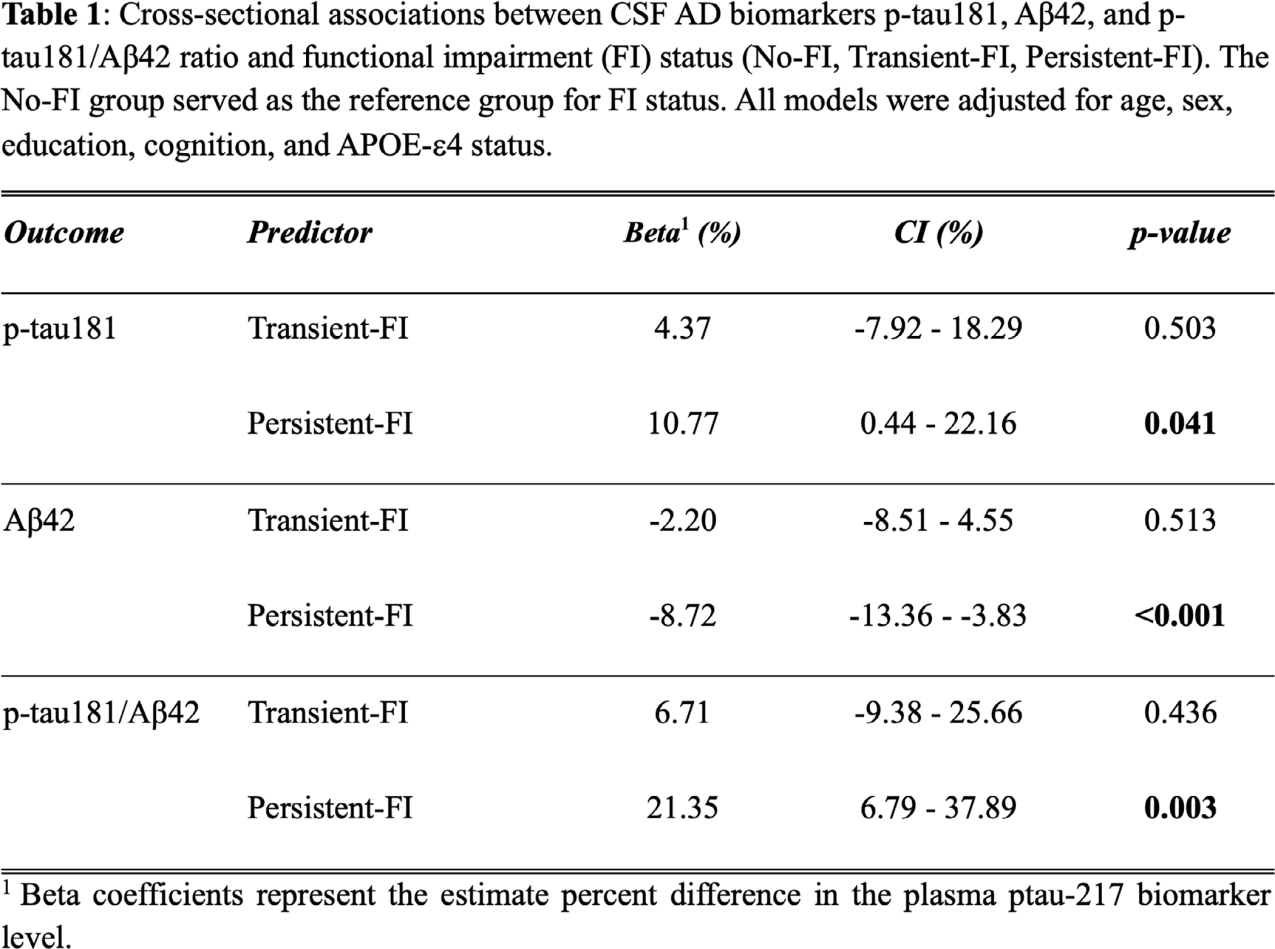# Functional impairment in association with biofluid biomarkers of Alzheimer's disease in dementia‐free older adults

**DOI:** 10.1002/alz70856_103852

**Published:** 2025-12-26

**Authors:** Maryam Ghahremani, Zahinoor Ismail

**Affiliations:** ^1^ Hotchkiss Brain Institute, University of Calgary, Calgary, AB, Canada; ^2^ University of Calgary, Calgary, AB, Canada

## Abstract

**Background:**

Dementia research is increasingly focused on developing methods for the early identification of individuals with underlying Alzheimer's disease (AD) pathology. Functional impairment (FI) is a key criterion for diagnosing dementia. However, subtle changes in function may occur during the preclinical and prodromal phases but are often not accurately characterized. Furthermore, there are few studies investigating the association between these subtle changes in functional abilities and AD biofluid biomarkers. Here we examined cross‐sectional associations between established cerebrospinal fluid (CSF) AD biomarkers and persistent versus transient FI in dementia‐free older adults.

**Method:**

Data from 1001 individuals (mean age 72.9±7.0; 45.2% female; 62.6% MCI; 41.5% APOE‐e4 carrier) from the Alzheimer's Disease Neuroimaging Initiative were analyzed. CSF biomarkers of interest included *p*‐tau181, amyloid‐beta42 (Aβ42), and the ptau‐181/Aβ42 ratio. Participants without baseline biomarker data were excluded. Using factor analysis, Functional Activities Questionnaire items of preparing meals, heating water to make warm beverages, and shopping were selected to quantify function. Persistent FI was operationalized as FI present at >two‐thirds of visits prior to dementia. Comparator groups included Transient‐FI and No‐FI. Linear regression modeled the association between FI status and biomarker levels at baseline, adjusting for age, sex, education, cognition, and APOE‐e4 status.

**Result:**

Compared to no FI, Persistent FI was associated with significantly higher *p*‐tau181 levels (Beta=10.77; CI[0.44‐22.16]; *p* = 0.041) and lower Aβ42 levels (Beta=‐8.72; 95%CI: [‐13.86‐ ‐3.83]; *p* <0.001) at baseline, while transient FI was not (*p*‐tau181: *p* = 0.503; Aβ42: *p* = 0.513). Similarly, Persistent FI was associated with a significantly higher ptau181/Aβ42 ratio (Beta=21.35; 95%CI: [6.79‐37.89]; *p* = 0.003), which demonstrated the greatest effect size among all models. Transient FI did not show a significant association (*p* = 0.436) (Table 1).

**Conclusion:**

Our cross‐sectional findings contribute to the limited research on the association of FI with CSF biomarkers of AD in dementia‐free older adults. Operationalizing FI‐related risk based on persistence enhances prognostication and identifies high‐risk individuals with greater burden of underlying AD pathology than those with transient FI or no FI. This approach may offer a more accurate method for early detection and stratification of at‐risk individuals, potentially guiding interventions before the onset of dementia.